# Effect of Chemical Solvents on the Wetting Behavior Over Time of Femtosecond Laser Structured Ti6Al4V Surfaces

**DOI:** 10.3390/nano10061241

**Published:** 2020-06-26

**Authors:** Georg Schnell, Christian Polley, Stephan Bartling, Hermann Seitz

**Affiliations:** 1 Microfluidics, Faculty of Mechanical Engineering and Marine Technology, University of Rostock, Justus-von-Liebig Weg 6, 18059 Rostock, Germany; christian.polley@uni-rostock.de (C.P.); hermann.seitz@uni-rostock.de (H.S.); 2 Leibniz Institute for Catalysis, Albert-Einstein-Straße 29a, 18059 Rostock, Germany; stephan.bartling@catalysis.de; 3 Department Life, Light & Matter, University of Rostock, Albert-Einstein-Str. 25, 18059 Rostock, Germany

**Keywords:** femtosecond laser, wetting, Ti6Al4V, cleaning, hydroxyl, organic groups, solvents, surfaces, interfaces, biomaterials

## Abstract

The effect of chemical solvents on the wetting state of laser-structured surfaces over time is systematically examined in this paper. By using a 300-fs laser, nanostructures were generated on Ti6Al4V, subsequently cleaned in an ultrasonic bath with different solvents and stored in ambient air. The static contact angle showed significant differences for cleaning with various solvents, which, depending on the applied cleaning and time, amounted up to 100°. X-ray photoelectron spectroscopy analyses reveal that the cleaning of the laser-structured surfaces affects the surface chemistry and the aging behavior of the surfaces, even with highly volatile solvents. The effect of the chemical surface modification is particularly noticeable when using alcohols for cleaning, which, due to their OH groups, cause highly hydrophilic behavior of the surface after one day of storage. Over the course of 14 days, enrichment with organic groups from the atmosphere occurs on the surface, which leads to poorer wetting on almost every structured surface. In contrast, the cleaning in hexane leads to a fast saturation of the surface with long-chain carbon groups and thus to a time-independent hydrophobic behavior.

## 1. Introduction

Bio-inspired functional surfaces have been of growing interest over the last decades and are receiving high attention in current research. One of the surface properties that is derived from nature is the ability to provide a (super-) hydrophilic or (super-) hydrophobic wetting behavior. For instance, the likeness of lotus leaves [1], rice leaves [2], the legs of water striders [3] and butterfly wings [4] are used in bionics to create surfaces with desired wetting properties for technical applications.

A changeable wettability of a surface offers additional, beneficial effects [5]. These are used in the design of self-cleaning surfaces [6,7], to improve corrosion [8,9], reduce bacteria attachment and to develop smart mechano-bactericidal surfaces [10,11,12]. Furthermore, the light absorption can be increased due to microstructures, which makes these surfaces useful for solar collectors [13,14]. The laser structuring is also very promising for biomedical products [15,16]. For such applications, the structuring of surfaces with short pulse laser or, in particular, ultra-short pulse lasers is of special importance in research and industrial applications. Due to the ability of creating stochastic (nano- and/or micro) [17,18,19] and highly precise deterministic structures [20,21] it is possible to initiate a desired wetting state in a fast processing time without subsequent processing on metals [22]. Therefore, the static contact angle (SCA) is often used to characterize the wetting state on laser-structured surfaces and related to relevant structuring parameters and biological response [23,24]. However, the wetting state is based on the chemical composition of the liquid and of the surface of the solids as well as the topography of the surface [25,26]. It was shown in several studies that the wetting behavior of laser structured surfaces on metals changes over time due to the adsorption of organic groups in ambient air or other organic rich atmospheres [16,27,28,29,30,31,32]. This aging process affects the chemical composition of the surface and it was shown that the phenomenon depends on the applied laser parameters [28], the material properties [33] and the storage conditions [27,29].

Table 1 lists exemplary publications with a focus on the adsorption of organic groups and on the change in wetting behavior. Often, ethanol, acetone, isopropyl and (ultra) pure or dionized water are used to remove impurities before the laser treatment (LT) or residues after LT. Further studies used acetone before LT [22,34,35,36], after LT [22,26,35,36,37] or cleaning with water followed by acetone [38]. The same applies for ethanol, which is used before [35,36,39,40] and after LT [35,36,41], and isopropanol, which is also used before [42] and after LT [43]. Especially in terms of biomedical applications alcohols play a special role, for disinfecting surfaces or for preparing in vitro and in vivo experiments [44]. Especially in implantology, LT is increasingly used and surface modification is correlated with surface wettability and cell reaction. However, often the effect of a cleaning agent on surface chemistry and surface wetting properties is not, or is only insufficiently, considered [45,46]. In summary, no consistent cleaning method exists and the effect of cleaning solvents on the change in wetting behavior has not been considered yet. Therefore, the effect of cleaning solvents on the aging process of laser structured surfaces should be elucidated. This study aims to investigate the interplay between laser surface structuring and surface chemistry affecting the surface wetting behavior depending on the cleaning process after laser irradiation on Ti6Al4V as a widely used material for biomedical application.

## 2. Materials and Methods

### 2.1. Materials and Cleaning Procedure

Ti6Al4V plates (10 × 10 × 4 mm) were purchased from S + D Spezialstahl Handelsgesellschaft mbH (Stelle, Germany) and used for all experimental investigations. To ensure a homogeneous and low roughness, the samples were polished with silicon carbide abrasive sandpaper from P320 (*t_1_* = 4 min), P600 (*t*_2_ = 4 min) to P1200 (*t*_3_ = 8 min) grain size before laser treatment. The resulting average area surface roughness of the samples were *Sa* 0.06 ± 0.005 µm. In the following, unstructured, polished and water rinsed samples serve as a reference. To purify the irradiated specimens, cleaning was performed in an ultrasonic bath (Sonorex Super RK 100/K, Bandelin Electronic GmbH & Co. KG, Berlin, Germany) to remove loose particles from the ablation process at 20 °C after LT with different cleaning solvents. All samples were structured separately and cleaned immediately after laser processing to maintain a constant delay between the end of LT and the cleaning step. The cleaning solvents were varied according to Table 2 to subsequently analyze the effect on the chemical composition of the surface and the effect on the resulting wetting state. In addition to the usual cleaning solvents mentioned above, hexane was used to further clarify the role of the chemical solvents. Drying was performed with dust-free wipes of the type KIMTECH Science precision wipes (Kimberly-Clark Global Sales, Roswell, NM, USA).

### 2.2. Laser Treatment

Specimens were structured using a fiber laser of the type UFFL _60_200_1030_SHG from Active Fiber Systems GmbH (Jena, Germany) with an amorphous glass Yb-doped core, a pulse duration of 300 fs and a wavelength of 1030 nm. The Gaussian laser beam is deflected in a horizontal direction by a scanner system of the type intelliSCANse (Scanlab GmbH, Puchheim, Germany). A F-theta lens with a focal length of 163 mm focuses the linear polarized laser light to a theoretical beam diameter of 36 microns on the sample surface. The system enables a repetition rate of 50.3 kHz up to 18.6 MHz with an average power of up to 60 W. The repetition rate was fixed at 226.8 kHz. The procedure for the laser processing is based on previous studies [16,19]. Both pulse and line overlap were 60% and a fluence of 2.46 J/cm^2^ was used for LT. The laser system is integrated into a 5-axis micromachining center Microgantry GU4 (Kugler GmbH, Salem, Germany). The laser structuring took place on an area of 7 × 7 mm under ambient air in a room with constant relative humidity and temperature (20 °C), to ensure equal treatment and comparability of different cleaned samples. The structures were produced nine times for each cleaning method, to avoid distorting through residuals of previous contact angle measurements.

### 2.3. Storage Conditions

Samples were stored under atmosphere in a laminar flow box of the type FBS (Spetec Gesellschaft für Labor- und Reinraumtechnik mbH, Erding, Germany) in a clean room equipped with UV-filtered light at 20 °C after laser irradiation and purification in the ultrasonic bath. Separate Petri dishes were used for storage of each sample and kept open at storage time.

### 2.4. Surface Wettability

Commonly used stable contact angle (SCA) measurements using the sessile drop method with a video-based optical contact angle measuring system OCA 40 Micro (DataPhysics Instruments GmbH, Filderstadt, Germany) were carried out after 1, 7 and 14 days in ambient air conditions at 20 °C. SCA measurements were repeated three times on separated areas for each point of time and carried out with a defined drop volume of 5 µL pure water with a defined dosage rate of 1 µL/s. A syringe Injekt-F Solo 9166017V (B. Braun Melsungen AG, Melsungen, Germany) with a cannula Sterican^®^ insulin G 27 × 1/2” (B. Braun Melsungen AG) was used. A picture was taken 3 s after the drop was dispensed. For calculation of the SCAs, the software Software SCA 20_U (Version 2, 2010, DataPhysics Instruments GmbH, Filderstadt, Germany) was used. The procedure the SCA measurements is based on previous studies [16,20].

### 2.5. Surface Characterization

For imaging of the topography and measuring the roughness of the surfaces, a confocal laser scanning microscope (CLSM) LEXT OLS 4000 and the software OLS4000 (Version 2.2.3, 2012, Olympus, Hamburg, Germany) were used. The average area surface roughness *Sa* was determined ten times at random areas for reference and structured sample, respectively. An optical magnification of 50× was used, leading to a scan area of 256 × 256 µm. A scanning electron microscope (SEM) Merlin VP compact (Carl Zeiss AG, Jena, Germany) was used to take high-resolution figures of the reference and structured surfaces.

### 2.6. Surface Chemical Analysis via XPS

XPS (X-ray photoelectron spectroscopy) elemental analysis was carried out 1 day and 14 days after LT to unravel the aging process of structured Ti6Al4V samples posterior storage due to chemical changes of the surface in ambient air. The XPS measurements were performed on an ESCALAB 220iXL (Thermo Fisher Scientific, Waltham, MA, USA) with a base pressure of 8 × 10^−10^ mbar using monochromated Al Kα radiation (E = 1486.6 eV). Samples are prepared on a stainless-steel holder with conductive double-sided adhesive carbon tape and placed inside the load lock (base pressure 3 × 10^−7^ mbar) for about 12 h prior to the measurement. The electron binding energies are referenced to the C 1s core level of carbon at 284.8 eV (C–C and C–H bonds). For quantitative analysis the peaks were deconvoluted with Gaussian-Lorentzian curves using the software Unifit 2020 (Unifit Scientific Software GmbH, Leipzig, Germany, 2019). The peak areas were normalized by the transmission function of the spectrometer and the element-specific sensitivity factor of Scofield [47].

## 3. Results and Discussion

### 3.1. Surface Topography

**Figure 1:**
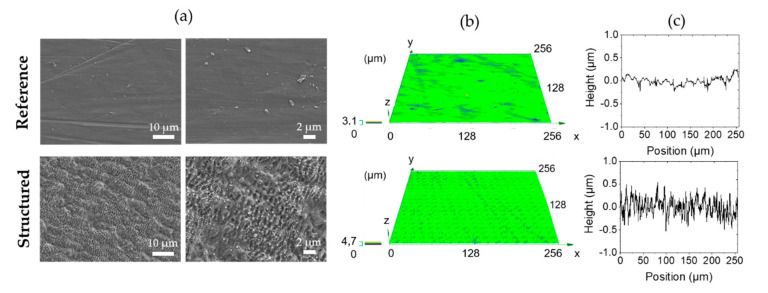
Surface characteristics of the reference and the structured surface. (**a**) SEM images (**b**) CLSM mapping and (**c**) elevation profile heights.

Figure 1 shows the surface characteristics of the reference and surface after fs laser irradiation. SEM images of the reference show the bright material with a large smooth surface and slight irregularities due to the polishing process. The SEM images of the structured surface suggest a heterogeneously composed surface modification. The structured surface is composed of femtosecond laser-induced periodic surface structures (FLIPSS) and melt displacement, as can be seen in Figure 1a,b. Both surface structures are demonstrated in many studies and the causes for the formation of nanostructures and melt displacement are widely understood [48,49]. Height elevations in Figure 1c confirm the modification of the surface towards the reference due to the formation of these nano structures. The differences in surface topography are reflected by the resulting average area surface roughness, whereby Sa for the reference is 0.06 ± 0.005 µm and for the structured surface amounts to 0.69 ± 0.09 µm. A detailed overview of the effects of similar laser parameters on the formation of nano- and microstructures on Ti6Al4V is given in [19].

### 3.2. Contact Angle Measurements

**Figure 2:**
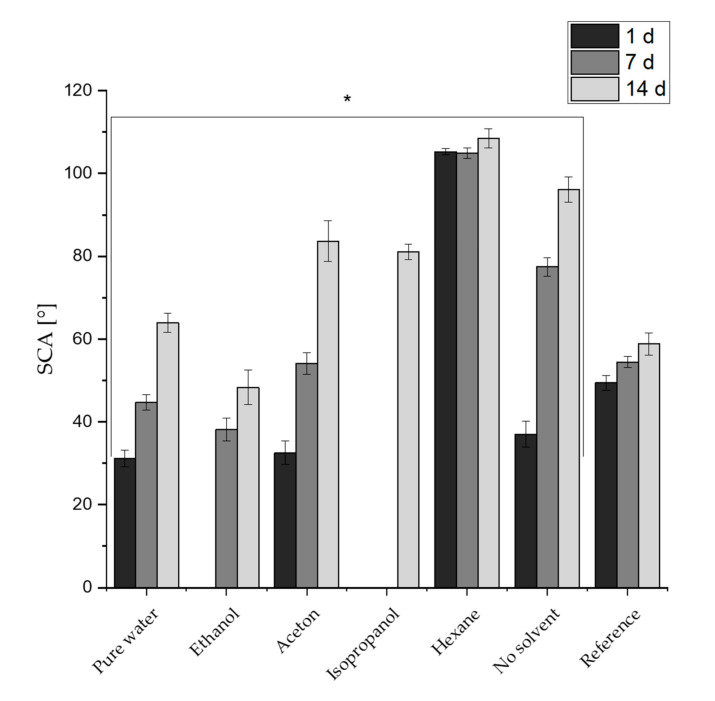
Static contact angles (SCA) on reference and structured specimens (indicated by (*)) as a function of time and used cleaning solvents.

Figure 2 displays the SCAs on the reference and structured specimens after 1 day, 7 and 14 days depending on the cleaning method. The SCAs of the reference surface show a slight increase of wetting behavior over time. The SCAs are 49.4 ± 1.9° after one day, 54.4 ± 1.4° after 7 days and 58.8 ± 2.6° after 14 days of storage. On the structured surfaces, a significant increase of SCAs on all structured specimens over time is evident. Independent of the cleaning method, all SCAs increase over the course of 14 days. After one day of storage, the SCAs for the surfaces cleaned with the alcohols were not measurable due to a superhydrophilic behavior of the surfaces. In contrast, the SCAs of the remaining cleaning solvents could be determined. The SCAs of the surfaces cleaned with pure water (31.1 ± 1.9°) and acetone (32.5 ± 2.6), as well as the uncleaned surface, are quite similar (37.0 ± 3.1°) after one day of storage, whereas the SCA for the hexane-cleaned surface is clearly higher (105.3 ± 0.7°). Therefore, the difference in the measured SCA after one day of storage on structured surfaces can be greater than 100° depending on the cleaning solvent used. Even after 7 and 14 days, the structured surface cleaned in hexane shows the highest SCA (108.5 ± 2.3°), followed by the SCA for the uncleaned structured surface (96.1 ± 3.2°), the SCA for the structure cleaned in acetone (83.7 ± 2.8°), the SCA for the structure cleaned in isopropanol (81 ± 1.9°), the SCA for the structure cleaned in pure water (61.9 ± 2.3°) and the SCA for the structure cleaned in ethanol (48.3 ± 4.2°). Therefore, the SCA for ethanol after 14 days of storage is clearly lower and shows the greatest difference in SCA measurement of 60.2° compared to the cleaning done in hexane. Therefore, a structured surface can be characterized as hydrophilic or hydrophobic depending on the cleaning solvent.

In summary, a clear dependence of the SCA on the cleaning used (or not used) solvent can be derived from the determined SCAs. Thus, since the manufacturing method and the roughness for all specimens is similar, it is obvious that the chemical composition of the solid interface plays a major role for the formation of the SCA.

### 3.3. Chemical Composition of the Surfaces and Its Effects on the Wetting State

**Figure 3:**
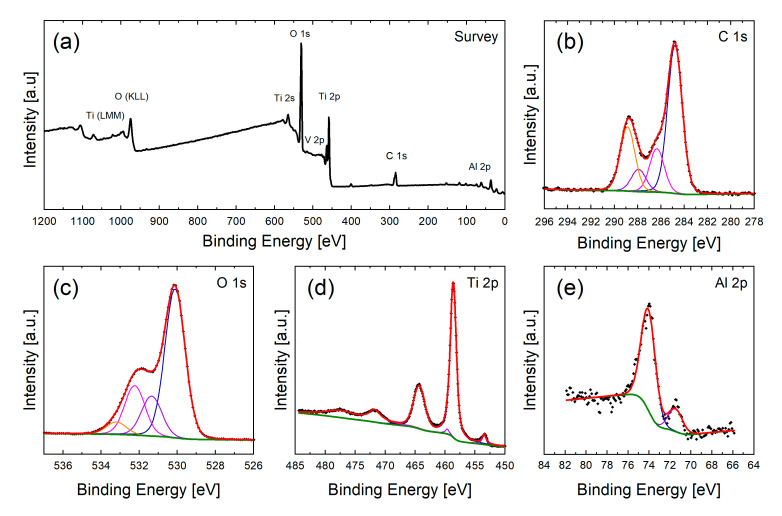
XP spectra of the unstructured sample without cleaning after 1d storage in air. In the survey spectra (**a**) the main components Ti, O, Al and C can be identified. The detailed high-resolution scans of these components C 1s (**b**), O 1s (**c**), Ti 2p (**d**) and Al 2p (**e**) are shown below.

**Figure 4:**
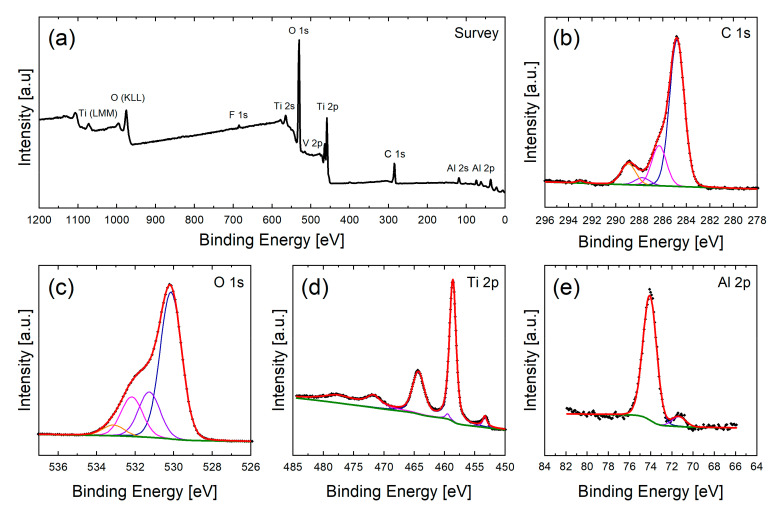
XP spectra of the structured sample without cleaning after 1d storage in air. This sample serves as a reference towards cleaned samples. In the survey spectra (**a**) the main components Ti, O, Al, C as well as small F contamination can be identified. The detailed high-resolution scans of C 1s (**b**), O 1s (**c**), Ti 2p (**d**) and Al 2p (**e**) are shown below.

XPS analyses were carried out to understand the effect of chemical cleaning with different cleaning solvents on the surface chemical composition and the resulting wetting behavior of fs laser structured samples. Figure 3 and Figure 4 show the XPS survey spectra and corresponding high-resolution scans of C 1s, O 1s, Ti 2p and Al 2p regions of the reference and the structured surface without cleaning after one day of storage in air, respectively. As can be seen from the survey spectra in Figure 3a and Figure 4a, the surface consists mainly of Ti and Al (as main compounds of Ti6Al4V) as well O and C, the latter mainly caused by organic groups deposited on the metal surface due to storage in air. Because of its low concentration as well as the overlap with the O 1s region the V 2p peaks are very weak. The surface also provides negligible amounts of N, F and Si which might originate in the sample handling (e.g., polishing and mounting for LT).

In the following, the main components Ti 2p, Al 2p, O 1s and C 1s are discussed in detail to elucidate the composition of the surface as well as changes through the cleaning and storage of the samples.

For this reason, the high-resolution spectra are deconvoluted with Gaussian-Lorentzian curves to determine the binding states of each component.

The C 1s spectrum (see Figure 3b and Figure 4b) is deconvoluted with four different carbon species. Characteristic binding energies are 284.8 eV for carbon-carbon (C–C) and hydrocarbon bonds (C–H), 286.3 eV carbon-oxygen (C–O), 287.7 eV for carbonyl (C=O) and 289.0 eV for carboxyl (O=C–O^−^) bonds [30,50].

The O 1s region can be described with four peaks (see Figure 3c and Figure 4c). Signals at about 530.2 eV and 531.2 eV are attributed to lattice oxygen of TiO_2_ [50] and Al_2_O_3_ as well as carbonyl groups (C=O) [30,51], respectively. A binding energy of about 532.1 eV is characteristic for carbon-oxygen bonds as well as hydroxyl groups adsorbed to the surface. Due to the same binding energy both groups cannot be distinguished unambiguously in the O 1s spectra [31,52] and will be described with one peak. Binding energies at about 533.1 eV can indicate the presence of phenol (C–OH) and/or ether (C–O–C) groups [51].

The high-resolution Ti 2p spectra (see Figure 3d and Figure 4d) indicates the presence of two oxidation states of titanium at the sample surface. The peaks at 458.6 eV and 464.3 eV correspond to the Ti 2p_3/2_ and Ti 2p_1/2_ signal of titanium in oxidation state of +IV [50,53]. The small peaks at 453.4 eV and 459.5 eV correspond to Ti 2p_3/2_ and Ti 2p_1/2_ of metallic Ti [50]. It is presumed that the oxide layer on top of the metal bulk material only has a thickness of a few nanometers so that the underlying metal can be seen by XPS.

This result is confirmed by the Al 2p spectrum (see Figure 3e and Figure 4e) which shows two main features at about 74.0 eV and 71.4 eV which can be identified as aluminum oxide (Al_2_O_3_) and metallic aluminum, respectively [29]. The oxide film thickness can be approximated using the metal:oxide ratio [54,55]. Assuming the correctness of this model for alloys an oxide film thickness of about 5 nm is estimated for the current system after polishing the surface and about 8 nm after LT.

The determined elements can be found in different amounts on all Ti6Al4V surfaces, both on the reference and the structured surface. To clarify the effect of cleaning procedures with cleaning solvents on the change in wetting behavior, it is necessary to trace the changes of surface chemistry in detail. In the past, other groups demonstrated that the change of surface chemistry over time on structured surfaces significantly alters the wetting properties of surfaces. Especially changes in the carbon and hydrocarbon composition were identified as a main reason for the transition from hydrophilic to hydrophobic wetting properties of metals [30]. It is known that the wetting state of a surface highly depends on the polarity of the investigated surface. A high polarity leads to a hydrophilic surface, whereas a low or non-polarity leads to hydrophobic wetting behavior on solid surfaces [56]. Carbon-oxygen bonds (C–O), carbonyl bonds (C=O) and O=C–O^−^ are classified as polar molecules whereas C–C and C–H are considered as non-polar compounds of a surface [29,30,57,58,59]. Therefore, in this study special attention is paid to the overall amount of carbon on the surface as well as long-chain alkylic C–C(H) compounds and the changes of the atomic ratio of C/(Al + Ti) on the surface to quantify the content of adsorbed carbon-rich organics [28,30].

**Figure 5:**
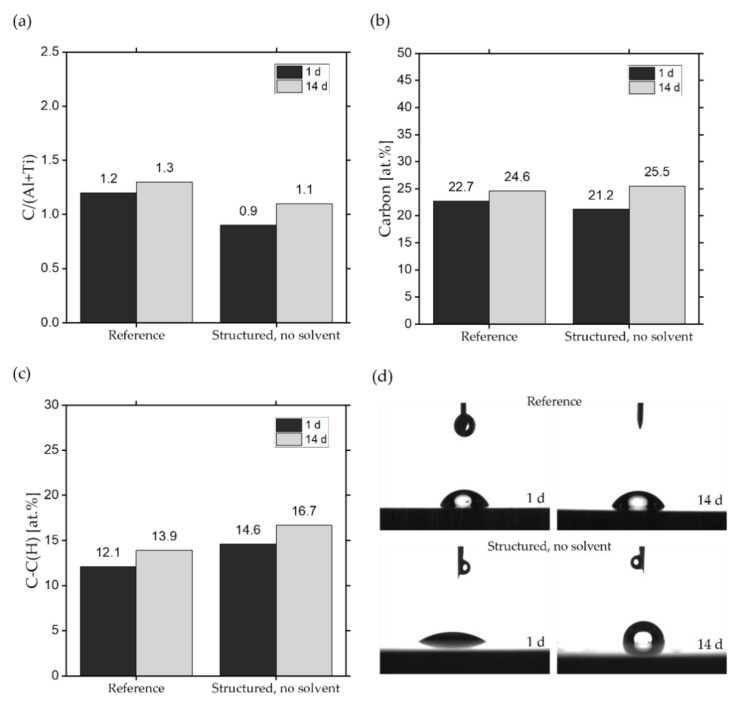
Comparison of the carbon content for the uncleaned structured surface and reference with exemplary images of dispensed droplets 1 and 14 days after storage. XPS data are shown for the (**a**) atomic ratio of C/(Al + Ti), (**b**) atomic carbon content and (**c**) atomic amount of C–C, C–H groups at 284.8 eV. Exemplary images of dispensed droplets on reference and structured surfaces without cleaning are shown in (**d**).

First, the effect of LT on the chemical composition of the surfaces without cleaning will be considered. To make differences in the chemical composition of the surface visible, changes of relevant compounds for the reference and the uncleaned structured surface are summarized in Figure 5. The change in polar compounds on the surface before and after LT was very little and therefore negligible, the results are shown in detail in the appendix (see Appendix A, Table A1).

The change in wetting behavior after laser structuring and storage of metals was demonstrated in earlier studies [27,28,29,30,31,32], revealing that laser treatment leads to removal of contaminations such as organic groups at the surface resulting in a lower C/Al ratio [27,28,29]. For the current system, the ratio C/(Al + Ti) was used to illustrate the cleaning effect of the laser treatment (see Figure 5a). Interestingly, the Al concentration at the surface increases after the laser structuring (see Appendix A Table A1 and Table 3) indicating enrichment of Al^3+^ in the top layers of the Ti6Al4V alloy. Al has a higher ablation threshold than Ti [60], which leads to different ablation rates and a different composition of the surface after LT. The higher portion of Al on the surface leads to a decrease of the C/(Al + Ti) ratio, which could be misinterpreted as a pronounced decrease of carbon on the surface. Looking at the total carbon concentration at the surface in Figure 5b only a small decrease is caused by the laser structuring (22.7% vs. 21.2%). However, this is a rather small change compared to other studies which might be correlated with the different pretreatment of the samples. As can be seen in Table 1, chemical solvents are often used before LT, which initially can saturate the surface with hydrocarbons, for example. Furthermore, the specimens in this study are polished to a bright material, contrary to all other studies listed in Table 1.

We assume that the main amount of contaminations had already been removed by the polishing process. Consequently, the resulting level of the carbon content on the surface of the reference and structured uncleaned surface after 1 day of storage in ambient air is quite comparable. Looking at the different contributions in the C 1s signal even an increase in the C–C(H) content (see Figure 5c) can be observed for the laser structured sample. Bearing in mind the slightly lower total carbon concentration after laser structuring, a removal of organic groups or transformation to simpler C–C, C–H bonds is assumed (see Appendix A, Table A1). As can be seen in Figure 5c, the portion of organic groups on the reference increases over time, leading to a slight increase of the SCA on the reference from 1 to 14 days after storage. This confirms the role of the organic groups on the formation of the SCA.

Table 3 indicates the formation of newly formed oxide, especially of alumina, compared to the reference due to the laser irradiation and exposure to air. It is known that the freshly formed titanium and aluminum oxide layers provide a large amount of unsaturated titanium, aluminum and oxygen, respectively. These oxide surfaces compounds function as Lewis acid and base sites, respectively, and are directly related to a highly hydrophilic surface due to a highly polar surface free energy [61,62]. In the following, hydroxylation of the surface took place very fast after laser irradiation due to the adsorption and subsequent dissociation of water molecules to the unsaturated elements (Al^3+^, Ti^4+^) from moisture in the ambient air. The reactivity with water decreases at the Lewis sites [27,61,63,64,65]. Therefore, we assume that the hydrophilic behavior of the structured sample without cleaning after 1 day of storage (see Figure 2) can be mainly explained by the higher quantity of unsaturated elements e.g., Al^3+^ content. Obviously, the immediate hydroxylation and the changed roughness of the surface also affect the resulting SCA after 1 day of storage. The impact on the surface roughness is well investigated and commonly the theories of Wenzel and Cassie-Baxter are applied to explain a full or partially wetted surface [66,67]. Therefore, the wetting state after laser irradiation can be characterized as wetting according to Wenzel’s theory.

For the subsequent gradual change of the wetting state over time on a structured surface (see SCAs of structured surface without cleaning in Figure 2), the adsorption of organic groups from ambient air was mentioned as the relevant phenomenon [30]. Especially the amount of alkylic groups, which are an essential part of almost all organic molecules, are supposed to evoke a more hydrophobic behavior, due to the intrinsic non-polar character. Whereas, pre-adsorbed hydroxyl groups act as primary binding sites and enable the chemisorption of non-polar groups. The rise of the amount of organic groups is indicated by the increase of the atomic content of C–C(H) from 14.6 (at.%) to 16.7 (at.%) and the atomic ratio of C/(Al + Ti) from 0.9 to 1.1 on the structured surface from 1 to 14 days of storage (see Figure 5). This leads to a transition of the wetting state and confirms the assumption in [30]. Despite the unchanged roughness of the structured sample, the surface is increasingly poorly wetted over time (see Figure 2) and the wetting state can be characterized as a partially wetted surface according to Cassis-Baxter. The adsorption of hydrocarbons can last for a long period of time depending on the storage conditions [28,29]. It is known that this increase in SCA can last for dozens of days when the samples are stored in air. This explains that the SCAs in this study are still increased even after 7 days of storage in ambient air (see Figure 2). The findings help to understand the effect of cleaning with solvents on the aging process of structured surfaces.

**Figure 6:**
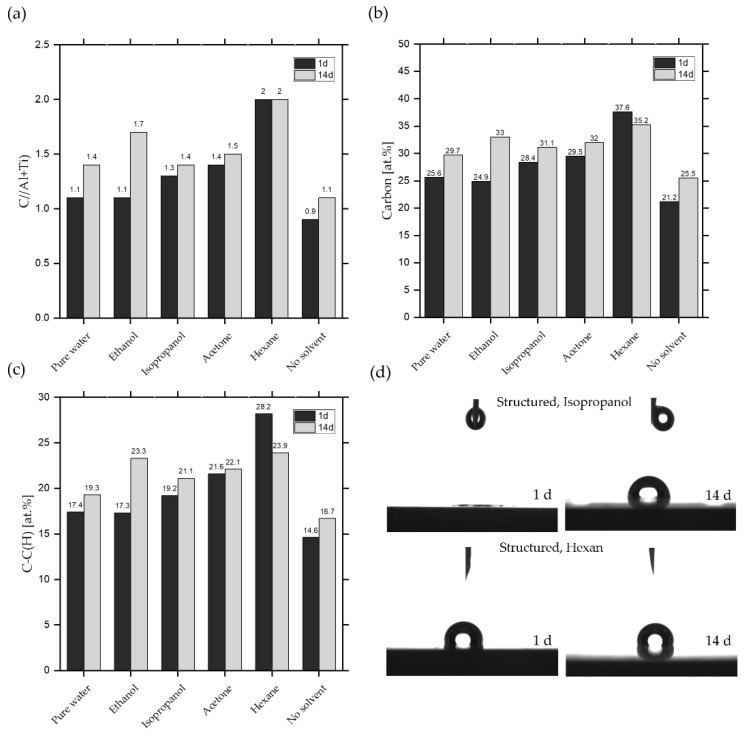
Comparison of the carbon content for structured surfaces and exemplary images of dispensed droplets for 1 and 14 days of storage. Structured surfaces are cleaned with different solvents and depicted in comparison to the uncleaned structured surface. XPS data are shown for the (**a**) atomic ratio C/(Al + Ti), (**b**) atomic total carbon content and (**c**) atomic amount of C–C(H) groups. Exemplary images of dispensed droplets on a structured surface cleaned in isopropanol and hexane for 1 and 14 days are shown in (**d**), respectively.

**Figure 7:**
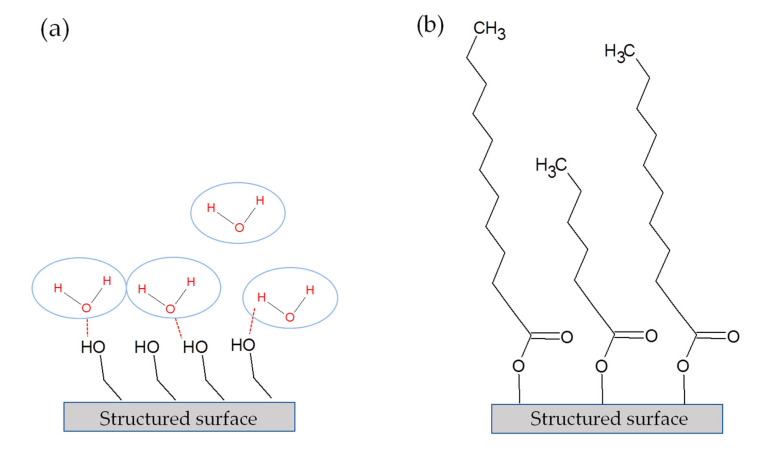
Exemplary schematic illustration of the adsorption of organic molecules on ethanol cleaned structured surface. (**a**) Structured surface immediately after cleaning. (**b**) Structured surface after a period of 14 days after cleaning.

**Figure A1:**
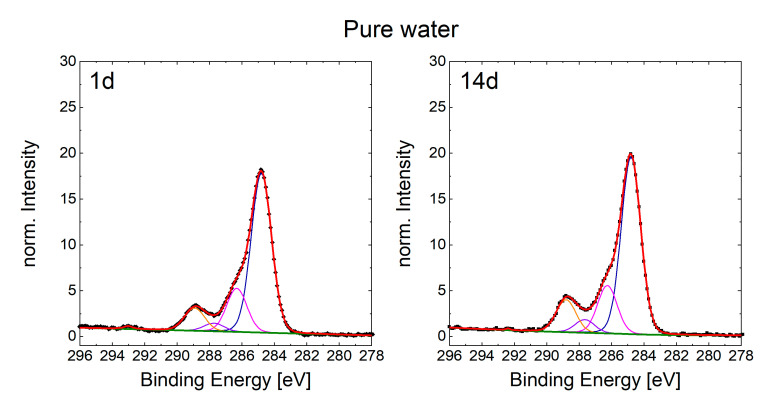
C 1s spectra of structured sample 1 and 14 days after cleaning with pure water.

**Figure A2:**
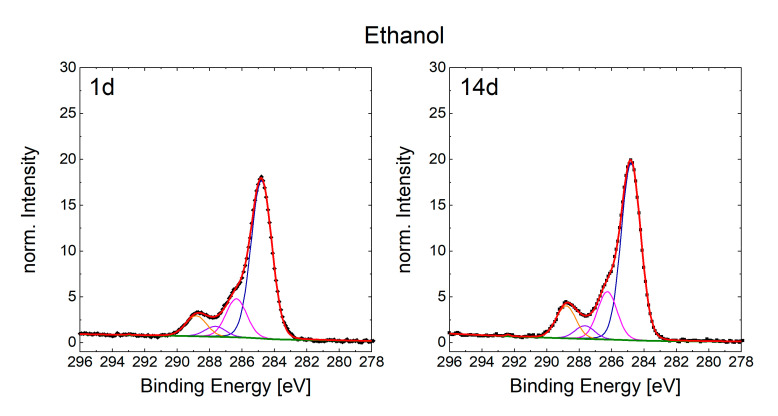
C 1s spectra of structured sample 1 and 14 days after cleaning with ethanol.

**Figure A3:**
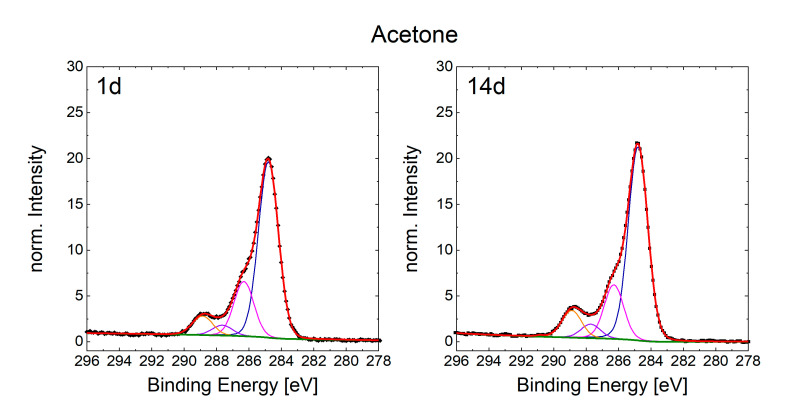
C 1s spectra of structured sample 1 and 14 days after cleaning with acetone.

**Figure A4:**
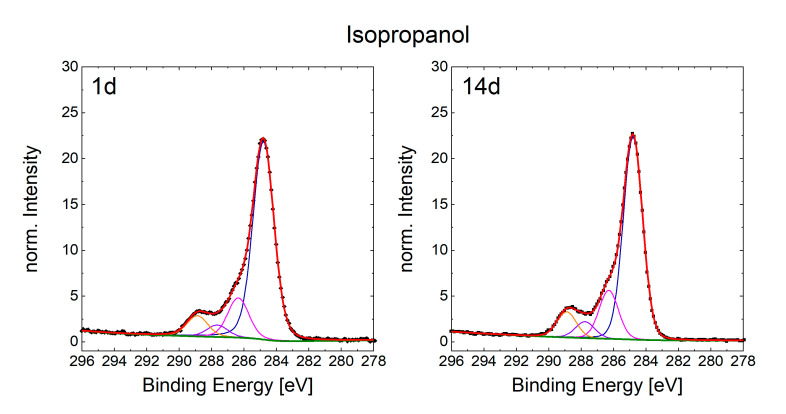
C 1s spectra of structured sample 1 and 14 days after cleaning with isopropanol.

**Figure A5:**
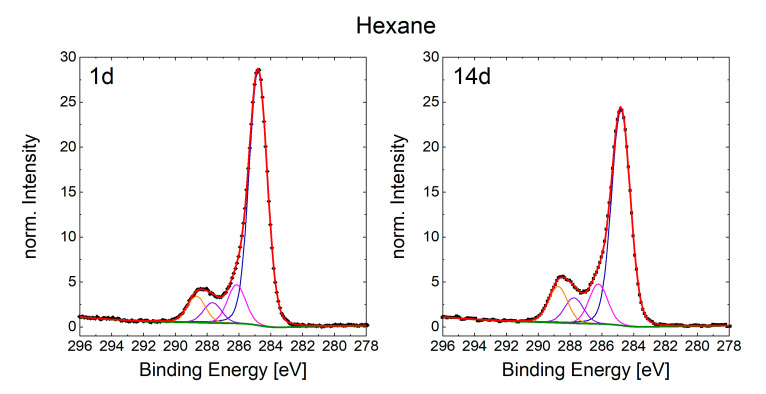
C 1s spectra of structured sample 1 and 14 days after cleaning with hexan.

**Figure A6:**
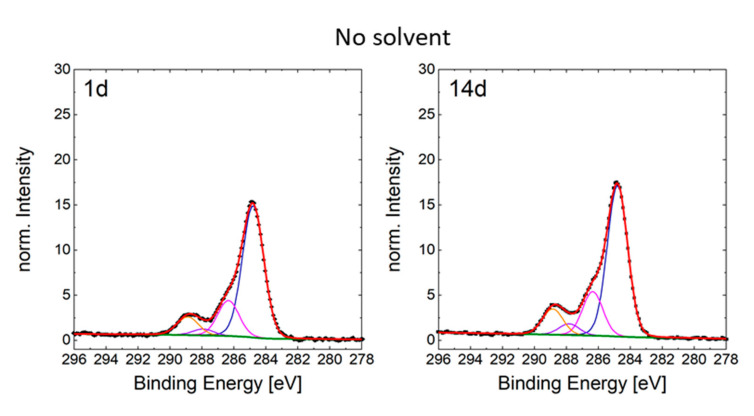
C 1s spectra of structured sample 1 and 14 days after preparation. No solvent was used for cleaning.

**Figure A7:**
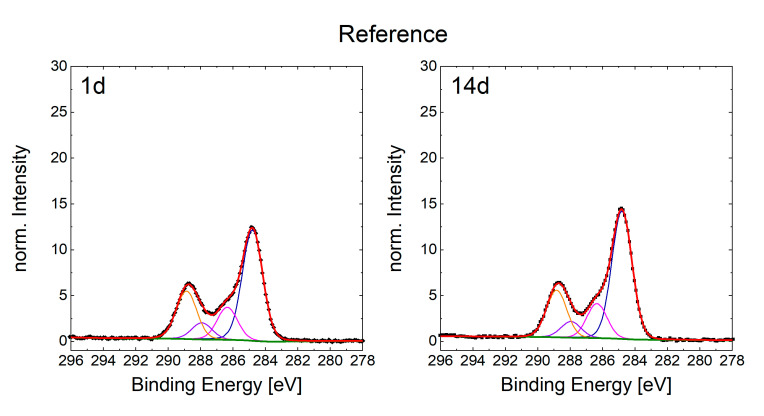
C 1s spectra of unstructured sample 1 and 14 days after preparation. No solvent was used for cleaning.

All structured surfaces cleaned in the ultrasonic bath show deviations in the C 1s spectra compared to the uncleaned structured surface (see Appendix B, Figure A1, Figure A2, Figure A3, Figure A4, Figure A5, Figure A6 and Figure A7 for C 1s high resolution spectra of all samples in the appendix). This concerns, in particular, the portion of C–C(H), the total amount of atomic carbon and the C/(Al + Ti) atomic ratio, as can be seen from the quantification data for C, Al and Ti in Figure 6. At first, it is noticeable that all cleaned surfaces provide more carbon, indicating that residuals of the cleaning solvents remain on the surface after drying, even with highly volatile fluids. Moreover, the deconvolution of the C 1s spectra revealed an uptake of carbon-rich compounds on the surface for all structured samples after 14 days, with the exception of the samples cleaned with hexane, which only shows little change. In this study, the C/(Al + Ti) ratio can be used to determine the amount of adsorbed organic groups over time for all structured surfaces, since the Al and Ti amount is rather stable (see Appendix A, Table A1). The XPS results correlate directly with the wetting properties of structured surfaces and confirm the increasing SCAs after 14 days of storage (Figure 2), due to the enrichment of carbon-rich compounds. Most striking is the cleaning with alcohols (ethanol, isopropanol), which shows a switch of wetting properties from super hydrophilic after one day to a hydrophilic or nearly hydrophobic wetting after 14 days of storage, respectively. We assume that the significant lower SCAs on alcohol-cleaned structured surfaces after one day is due to the fundamental components of OH^−^ groups in alcohols that remain on the structured surface after drying. OH^−^ groups referred as high polar compounds and cause an increase of van der Waals forces and hydrogen bond of OH^−^ and H_2_O [68,69,70]. Based on the XPS data, these groups cannot be clearly distinguished from the organic components as mentioned above. Since alcohols are also hydrocarbons, residues of the alcohols are also detected in XPS analysis. But OH^−^ groups can only be found in the applied alcohols and all other molecules (H, O, CH_3_) are also components of the other solvents used (see Table 2), thus confirming this thesis. It can be assumed that after cleaning, a nanometer-thick layer of alcohol molecules remains on the structured surface which induces super hydrophilic wetting properties due to the hydroxyl groups which form hydrogen bonds with water molecules. This mechanism is shown systematically in Figure 7a. Acetone and water have basically no OH^−^ groups in their chemical composition. Therefore, the cleaning of the structured surfaces in both solvents shows relatively similar SCAs compared to the uncleaned structured sample after 1 day of storage. After 14 days of storage, the structures cleaned in alcohols change their wetting behavior towards a poorer wetting which can be attributed to the bonding of hydrocarbons, carboxylic acids, and similar compounds from ambient air to the OH^−^ groups of the alcohols. Thus, the surface is covered with atmospheric carbon-rich organic molecules which replace the water and bind to the hydroxylic groups on the surface (Figure 7b). This aligns with the theory that hydroxylation of structured surfaces, through the reaction of coordinatively unsaturated metal ions (e.g., Al^3+^) with water, is necessary to create binding sites for hydrocarbons from the air [28,30,71]. The structured surfaces cleaned in pure water or acetone are also still reactive after cleaning and can adsorb organic compounds due to the mentioned process, which is represented by the rise of carbon groups for both in Figure 6. Whereas the SCAs for the pure water and the acetone cleaned sample after 14 days of storage do not correspond with the SCA of the uncleaned structured surface. 

The carbon amounts in Figure 6 alone cannot justify this behavior, since no clear correlation between the amount of C–C(H) groups and maximum SCAs caused by pure water or acetone compared to the uncleaned surface can be established. The adsorption of organic compounds from ambient air after laser irradiation is a complex phenomenon due to the huge diversity of molecules and groups occurring in ambient air, which results in an even more manifold chemical composition of cleaned surfaces. It must be assumed that residues from cleaning in water or acetone either affect the adsorption of organic groups or the residues themselves affect the SCA measurement. But the relative atomic carbon amount does not reach the same level of adsorbed non-polar groups compared to the cleaning with hexane. Organic compounds achieved from the ambient air are mostly medium or short-chain alkyls [30,72,73], which explains the lower level of C–C(H) in Figure 6c and the lower SCAs in comparison to the hexane-cleaned samples.

Hexane leads to the highest SCA values of all cleaning solvents containing non-polar groups and essentially results in the highest obtained SCAs. Hexane belongs to the long-chained alkanes and therefore shows high C–C(H) values in the XPS data in Figure 6c. Whereas hexane is a highly volatile liquid, the hydrocarbons appear to accumulate on the surface and form a permanent bond. The structured surface becomes saturated with carbon-rich, non-polar molecules which ultimately leads to a hydrophobic wetting state. The XPS results confirm the theory, showing an immediate high content of carbon on the surface, only with a minor change in two weeks. Similar behavior of the wetting properties of structured surfaces was observed in literature when structured surfaces were brought into an organic rich atmosphere. The surfaces showed the highest percentage of C–C(H) groups and the highest C/Al ratio after a short time [30]. The investigated structured surfaces in this study that were cleaned with hexane display a very similar behavior and are the ones with the highest percentage of carbon-rich compounds on the surface, reflected in the amount of C–C(H) and the C/(Al + Ti) ratio, compared to all other cleaning agents (see Figure 6a,c). After 14 days of storage, the carbonaceous content on the surface did not change significantly, which can be explained by the saturation of all binding sites on the metallic surface.

## 4. Conclusions

Static contact angle measurement (SCA) and X-ray photoelectron spectroscopy (XPS) analysis were carried out to clarify the role of different chemical solvents on the wetting behavior of femtosecond laser structured Ti6Al4V surfaces. The topography of the resulting surfaces was characterized by scanning electron microscope (SEM) and surface area roughness via confocal laser scanning microscopy (CLSM). The findings can be summarized as follows:

- The wetting behavior of laser structured surfaces clearly depends on the used cleaning solvent.
- Alcohols possess OH^−^ groups that affect the composition of structured surfaces and evoke a temporary hydrophilic behavior after laser treatment.
- The cleaning in hexane provides the highest SCAs after laser treatment within 14 days after laser treatment due to the initial adsorption of long-chained hydrocarbons.
- The cleaning of the surfaces after laser irradiation plays a major role in the formation of the SCA. Thus, SCAs should only be compared to each other where identical post-processing methods have been applied.
- The effect of cleaning with different solvents and adsorption of carbon on the formation of the SCA should be considered for all correlations between surface and secondary effects.

This study confirms the assumptions from the literature on the adsorption of organic molecules on laser-structured surfaces and provides information on the significant effect of cleaning solvents on the ageing process of the surfaces. Future studies should investigate the effect of chemical solvents under different storage conditions and consider the complete saturation of the surface after a long period of storage. An analysis of the advancing or receding contact angle should also be performed with differently cleaned samples over time.

## Figures and Tables

**Table 1:** Comparison of the usage of chemical solvents before and after laser treatment (LT) on metals in exemplary studies with focus on chemical altering of structured surfaces. US indicates the usage of an ultrasonic bath for cleaning of the samples before or after LT.

Material	Laser Pulse Duration	Cleaning before LT	Atmosphere at LT	Cleaning after LT	Storage after LT	Reference
Al2024	15 ns	Isopropanol	Ambient air	Compressed air	Ambient air, polyethylene bags and polystyrene boxes	[27]
Ti6Al4V	30 ns and 310 fs	Ethanol	Ambient air	Compressed air	Ambient air; polyethylene bags	[29]
Al	30 ns	No data (maybe acetone as typical degreasing agent for Al-foils)	Ambient air	No data	Ambient air	[28]
Ti6Al4V	30 ns	No data	Ambient air	No data	High vacuum	[31]
Al	10 ps	Ethanol in US	Ambient air	Ethanol + US, Compressed air	CO_2_, O_2,_ N_2_	[30]
Al	50 ns	acetone, ethanol and deionized water in sequence + US	No data	No data	Ambient Air; Annealing	[32]

**Table 2:** Cleaning methods after LT. Used cleaning solvents (Carl Roth GmbH + Co. KG, Karlsruhe, Germany) with structural chemical formula, purity and time of cleaning in ultrasonic bath. For pure water, instead of purity, conductivity is indicated and highlighted by *.

Cleaning Solvent	Structural Chemical Formula	Purity (%) (* Conductivity (µS/cm))	Time of Cleaning (min)
No cleaning	-	-	-
Pure water	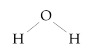	0.055 *	15
Ethanol	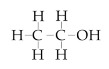	≥99.8	15
Acetone	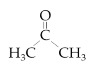	≥99.7	15
Isopropanol	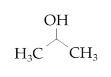	≥99.5	15
Hexane	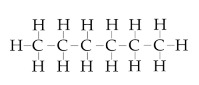	≥95	15

**Table 3:** Comparison of the relative amount of Al^0^, Al^3+^ and Ti^0^, Ti^4+^ on the surface of reference and structured surface without cleaning after 1 day of storage.

Specimens	Al^0^ (at. %)	Al^3+^ (at. %)	Ti^0^ (at. %)	Ti^4+^ (at. %)
Reference	0.7	3.2	0.7	15.2
Structured (no solvent)	0.7	8.8	1.0	14.2

## Abbreviations

LT	laser treatment
SCA	static contact angle
US	ultrasonic bath

## Appendix A

**Table A1:** Detailed summary of the XPS quantification data.

		Structured												Reference	
	Cleaning	Pure Water		Ethanol		Acetone		Isopropanol		Hexane		No Solvent		No Solvent	
	Time of storage (days)	1	14	1	14	1	14	1	14	1	14	1	14	1	14
Al 2p	Al^3+^ (at.%)	8.05	8.21	8.38	7.54	8.05	7.77	7.95	7.79	6.81	7.06	8.83	8.65	3.15	3.08
	Al^0^ (at.%)	0.61	0.69	0.66	0.42	0.53	0.5	0.61	0.4	0.59	0.5	0.65	0.59	0.69	0.66
	Al Total (at.%)	8.66	8.9	9.04	7.96	8.58	8.27	8.56	8.19	7.4	7.56	9.48	9.24	3.84	3.74
C 1s	C–C/C– (at.%)	17.36	19.32	17.25	23.26	19.19	21.11	21.61	22.1	28.19	23.94	14.56	16.66	12.1	13.95
	–C–OH (at.%)	4.72	5.27	4.24	5.14	5.96	5.66	4.34	5.3	4.29	4.42	3.9	4.81	3.56	3.75
	–C=O (at.%)	0.88	1.45	1.14	1.5	1.13	1.47	1.3	1.79	2.25	2.82	0.69	1.23	1.83	1.75
	O=CO– (at.%)	2.46	3.56	2.25	3.09	2.1	2.84	2.26	2.81	2.91	4.02	2.01	2.83	5.24	5.1
	Pi–Pi (at.%)	0.19	0.05	0.01	0	0	0.05	0	0	0	0	0	0	0	0
	C Total (at.%)	25.61	29.65	24.89	32.99	28.38	31.13	29.51	32.00	37.64	35.20	21.16	25.53	22.73	24.55
O 1s	TiO_2_ (at.%)	31.2	27.83	31.75	27.5	29.22	27.38	30.2	27.25	25.73	25.74	33.07	30.8	34.21	32.35
	Al_2_O_3_/C=O (at.%)	9.67	10.34	10.04	9.09	10.06	9.02	8.98	8.79	9.41	9.35	10.27	10.45	9.14	9.02
	O–C/OH– (at.%)	8.43	9.26	8.03	8.62	8.19	8.19	7.71	7.83	6.9	9.42	8.63	8.59	11.35	11.36
	C–O–C/C–OH (at.%)	2.25	2.13	2.19	2,25	2.1	2.12	1.94	2.19	1.43	2.33	2.26	1.98	2.82	3.17
	O Total (at.%)	51.55	49.56	52.01	47.46	49.57	46.71	48.83	46.06	43.47	46.84	54.23	51.82	57.52	55.9
Ti 2p	Ti^4+^ (at.%)	13.4	11.42	13.24	11.04	12.53	13.13	12.35	12.98	10.61	9.71	14.18	12.78	15.19	15.07
	Ti^0^ (at.%)	0.77	0.49	0.82	0.53	0.93	0.77	0.74	0.78	0.87	0.67	0.95	0.63	0.72	0.73
	Ti Total (at.%)	14.17	11.91	14.06	11.57	13.46	13.9	13.09	13.76	11.48	10.38	15.13	13.41	15.91	15.8

## References

[1] Barthlott W., Neinhuis C. (1997). Purity of the sacred lotus, or escape from contamination in biological surfaces. Planta.

[2] Feng L., Li S., Li Y., Li H., Zhang L., Zhai J., Song Y., Liu B., Jiang L., Zhu D. (2002). Super-hydrophobic surfaces: From natural to artificial. Adv. Mater..

[3] Gao X., Jiang L. (2004). Water-repellent legs of water striders. Nature.

[4] Zheng Y., Gao X., Jiang L. (2007). Directional adhesion of superhydrophobic butterfly wings. Soft Matter.

[5] Shin S., Seo J., Han H., Kang S., Kim H., Lee T. (2016). Bio-inspired extreme wetting surfaces for biomedical applications. Materials.

[6] Parkin I.P., Palgrave R.G. (2005). Self-cleaning coatings. J. Mater. Chem..

[7] Ganesh V.A., Raut H.K., Nair A.S., Ramakrishna S. (2011). A review on self-cleaning coatings. J. Mater. Chem..

[8] Kietzig A.-M., Hatzikiriakos S.G., Englezos P. (2009). Ice friction: The effects of surface roughness, structure, and hydrophobicity. J. Appl. Phys..

[9] Boinovich L.B., Emelyanenko A.M., Modestov A.D., Domantovsky A.G., Emelyanenko K.A. (2015). Synergistic Effect of superhydrophobicity and oxidized layers on corrosion resistance of aluminum alloy surface textured by nanosecond laser treatment. ACS Appl. Mater. Interfaces.

[10] Chaudhari A.A., Coppage-Gross J., Rangan K.K., Onyilo V.C., Sudarshan T.S., Singh S.R., Pillai S.R. (2016). Multifunctionally modified superhydrophobic aluminum and fabric surfaces with reduced gram-negative and gram-positive bacterial attachment: A possible approach for self-cleaning aircraft and crew cabin surfaces. Mater. Manuf. Process..

[11] Linklater D.P., Juodkazis S., Crawford R.J., Ivanova E.P., Crawford R. (2019). Mechanical inactivation of Staphylococcus aureus and Pseudomonas aeruginosa by titanium substrata with hierarchical surface structures. Materialia.

[12] Ivanova E.P., Linklater D.P., Werner M., Baulin V.A., Xu X., Vrancken N., Rubanov S., Hanssen E., Wandiyanto J., Truong V.K. (2020). The multi-faceted mechano-bactericidal mechanism of nanostructured surfaces. Proc. Natl. Acad. Sci. USA.

[13] Vorobyev A.Y., Guo C. (2015). Multifunctional surfaces produced by femtosecond laser pulses. J. Appl. Phys..

[14] Hwang T.Y., Vorobyev A.Y., Guo C. (2011). Enhanced efficiency of solar-driven thermoelectric generator with femtosecond laser-textured metals. Opt. Express.

[15] Vorobyev A.Y., Guo C. (2007). Femtosecond laser structuring of titanium implants. Appl. Surf. Sci..

[16] Schnell G., Staehlke S., Duenow U., Nebe J.B., Seitz H. (2019). Femtosecond laser nano/micro textured Ti6Al4V surfaces-effect on wetting and MG-63 cell adhesion. Materials.

[17] Oliveira V., Ausset S., Vilar R. (2009). Surface micro/nanostructuring of titanium under stationary and non-stationary femtosecond laser irradiation. Appl. Surf. Sci..

[18] Moradi S., Kamal S., Englezos P., Hatzikiriakos S.G. (2013). Femtosecond laser irradiation of metallic surfaces: Effects of laser parameters on superhydrophobicity. Nanotechnology.

[19] Schnell G., Duenow U., Seitz H. (2020). Effect of laser pulse overlap and scanning line overlap on femtosecond laser-structured Ti6Al4V surfaces. Materials.

[20] Schnell G., Jagow C., Springer A., Frank M., Seitz H. (2019). Time-dependent anisotropic wetting behavior of deterministic structures of different strut widths on Ti6Al4V. Metals.

[21] Wang C., Hu H., Li Z., Shen Y., Xu Y., Zhang G., Zeng X., Deng J., Zhao S., Ren T. (2019). Enhanced osseointegration of titanium alloy implants with laser microgrooved surfaces and graphene oxide coating. ACS Appl. Mater. Interfaces.

[22] Rukosuyev M.V., Lee J., Cho S.J., Lim G., Jun M.B.G. (2014). One-step fabrication of superhydrophobic hierarchical structures by femtosecond laser ablation. Appl. Surf. Sci..

[23] Schlie S., Fadeeva E., Koch J., Ngezahayo A., Chichkov B.N. (2010). Femtosecond laser fabricated spike structures for selective control of cellular behavior. J. Biomater. Appl..

[24] Fadeeva E., Schlie S., Koch J., Ngezahayo A., Chichkov B.N. (2009). The hydrophobic properties of femtosecond laser fabricated spike structures and their effects on cell proliferation. Phys. Status Solidi (a).

[25] Guan Y.C., Luo F.F., Lim G.C., Hong M.H., Zheng H.Y., Qi B. (2015). Fabrication of metallic surfaces with long-term superhydrophilic property using one-stop laser method. Mater. Des..

[26] Kietzig A.-M., Hatzikiriakos S.G., Englezos P. (2009). Patterned superhydrophobic metallic surfaces. Langmuir.

[27] Cardoso J.T., Garcia-Girón A., Romano J.M., Huerta-Murillo D., Jagdheesh R., Walker M., Dimov S.S., Ocaña J.L. (2017). Influence of ambient conditions on the evolution of wettability properties of an IR-, ns-laser textured aluminium alloy. RSC Adv..

[28] Jagdheesh R., Diaz M., Ocaña J.L. (2016). Bio inspired self-cleaning ultrahydrophobic aluminium surface by laser processing. RSC Adv..

[29] Huerta-Murillo D., García-Girón A., Romano J.M., Cardoso J.T., Cordovilla F., Walker M., Dimov S.S., Ocaña J.L. (2019). Wettability modification of laser-fabricated hierarchical surface structures in Ti6Al4V titanium alloy. Appl. Surf. Sci..

[30] Long J., Zhong M., Zhang H., Fan P. (2015). Superhydrophilicity to superhydrophobicity transition of picosecond laser microstructured aluminum in ambient air. J. Colloid Interface Sci..

[31] Jagdheesh R., Diaz M., Marimuthu S., Ocaña J.L. (2019). Hybrid laser and vacuum process for rapid ultrahydrophobic Ti6Al4 V surface formation. Appl. Surf. Sci..

[32] Yang Z., Liu X., Tian Y. (2019). Insights into the wettability transition of nanosecond laser ablated surface under ambient air exposure. J. Colloid Interface Sci..

[33] Takeda S., Fukawa M., Hayashi Y., Matsumoto K. (1999). Surface OH group governing adsorption properties of metal oxide films. Thin Solid Films.

[34] Jagdheesh R., Pathiraj B., Karatay E., Römer G.R.B.E., Huis in’t Veld A.J. (2011). Laser-induced nanoscale superhydrophobic structures on metal surfaces. Langmuir.

[35] Bizi-Bandoki P., Valette S., Audouard E., Benayoun S. (2013). Time dependency of the hydrophilicity and hydrophobicity of metallic alloys subjected to femtosecond laser irradiations. Appl. Surf. Sci..

[36] Lee B.E.J., Exir H., Weck A., Grandfield K. (2018). Characterization and evaluation of femtosecond laser-induced sub-micron periodic structures generated on titanium to improve osseointegration of implants. Appl. Surf. Sci..

[37] Cunha A., Serro A.P., Oliveira V., Almeida A., Vilar R., Durrieu M.-C. (2013). Wetting behaviour of femtosecond laser textured Ti6Al4V surfaces. Appl. Surf. Sci..

[38] May A., Agarwal N., Lee J., Lambert M., Akkan C.K., Nothdurft F.P., Aktas O.C. (2015). Laser induced anisotropic wetting on Ti6Al4V surfaces. Mater. Lett..

[39] Bizi-Bandoki P., Benayoun S., Valette S., Beaugiraud B., Audouard E. (2011). Modifications of roughness and wettability properties of metals induced by femtosecond laser treatment. Appl. Surf. Sci..

[40] Lu J., Huang T., Liu Z., Zhang X., Xiao R. (2018). Long-term wettability of titanium surfaces by combined femtosecond laser micro/nano structuring and chemical treatments. Appl. Surf. Sci..

[41] He A., Liu W., Xue W., Yang H., Cao Y. (2018). Nanosecond laser ablated copper superhydrophobic surface with tunable ultrahigh adhesion and its renewability with low temperature annealing. Appl. Surf. Sci..

[42] Milles S., Voisiat B., Nitschke M., Lasagni A.F. (2019). Influence of roughness achieved by periodic structures on the wettability of aluminum using direct laser writing and direct laser interference patterning technology. J. Mater. Process. Technol..

[43] Zupančič M., Može M., Gregorčič P., Golobič I. (2017). Nanosecond laser texturing of uniformly and non-uniformly wettable micro structured metal surfaces for enhanced boiling heat transfer. Appl. Surf. Sci..

[44] DIN EN ISO 10993-5:2009-10 (2009). Biologische Beurteilung von Medizinprodukten – Teil-5: Prüfungen auf In-vitro-Zytotoxizität (ISO_10993-5:2009).

[45] Fadeeva E., Deiwick A., Chichkov B., Schlie-Wolter S. (2014). Impact of laser-structured biomaterial interfaces on guided cell responses. Interface Focus.

[46] Ranella A., Barberoglou M., Bakogianni S., Fotakis C., Stratakis E. (2010). Tuning cell adhesion by controlling the roughness and wettability of 3D micro/nano silicon structures. Acta Biomater..

[47] Scofield J.H. (1976). Hartree-Slater subshell photoionization cross-sections at 1254 and 1487 eV. J. Electron. Spectrosc. Relat. Phenom..

[48] Vorobyev A.Y., Guo C. (2013). Direct femtosecond laser surface nano/microstructuring and its applications. Laser Photonics Rev..

[49] Cristoforetti G., Legnaioli S., Palleschi V., Tognoni E., Benedetti P.A. (2008). Observation of different mass removal regimes during the laser ablation of an aluminium target in air. J. Anal. At. Spectrom..

[50] Biesinger M.C., Lau L.W.M., Gerson A.R., Smart R.S.C. (2010). Resolving surface chemical states in XPS analysis of first row transition metals, oxides and hydroxides: Sc, Ti, V, Cu and Zn. Appl. Surf. Sci..

[51] Plomp A.J., Su D.S., de Jong K.P., Bitter J.H. (2009). On the Nature of Oxygen-Containing Surface Groups on Carbon Nanofibers and Their Role for Platinum Deposition—An XPS and Titration Study. J. Phys. Chem. C.

[52] Wulser K.W., Langell M.A. (1992). Carboxylic acid adsorption on NiO(100) characterized by X-ray photoelectron and high resolution electron energy loss spectroscopies. Catal. Lett..

[53] Buchholz M. (2013). UHV-FTIRS-Untersuchungen an einkristallinen Oxidoberflächen.

[54] Strohmeier B.R. (1990). An ESCA method for determining the oxide thickness on aluminum alloys. Surf. Interface Anal..

[55] Carlson T.A., McGuire G.E. (1972). Study of the x-ray photoelectron spectrum of tungsten—Tungsten oxide as a function of thickness of the surface oxide layer. J. Electron Spectrosc. Relat. Phenom..

[56] Giovambattista N., Debenedetti P.G., Rossky P.J. (2007). Effect of surface polarity on water contact angle and interfacial hydration structure. J. Phys. Chem. B.

[57] Wang Z.K., Zheng H.Y., Lim C.P., Lam Y.C. (2009). Polymer hydrophilicity and hydrophobicity induced by femtosecond laser direct irradiation. Appl. Phys. Lett..

[58] Lai J., Sunderland B., Xue J., Yan S., Zhao W., Folkard M., Michael B.D., Wang Y. (2006). Study on hydrophilicity of polymer surfaces improved by plasma treatment. Appl. Surf. Sci..

[59] Pandiyaraj K.N., Selvarajan V., Deshmukh R.R., Gao C. (2009). Modification of surface properties of polypropylene (PP) film using DC glow discharge air plasma. Appl. Surf. Sci..

[60] Mildner J., Sarpe C., Götte N., Wollenhaupt M., Baumert T. (2014). Emission signal enhancement of laser ablation of metals (aluminum and titanium) by time delayed femtosecond double pulses from femtoseconds to nanoseconds. Appl. Surf. Sci..

[61] Gentleman M.M., Ruud J.A. (2010). Role of hydroxyls in oxide wettability. Langmuir.

[62] Van Oss C.J., Good R.J., Chaudhury M.K. (1988). Additive and nonadditive surface tension components and the interpretation of contact angles. Langmuir.

[63] Eng P.J., Trainor T.P., Brown G.E., Waychunas G.A., Newville M., Sutton S.R., Rivers M.L. (2000). Structure of the hydrated alpha-Al(2)O(3) (0001) surface. Science.

[64] Van den Brand J., van Gils S., Beentjes P.C.J., Terryn H., de Wit J.H.W. (2004). Ageing of aluminium oxide surfaces and their subsequent reactivity towards bonding with organic functional groups. Appl. Surf. Sci..

[65] Tong S.R., Wu L.Y., Ge M.F., Wang W.G., Pu Z.F. (2010). Heterogeneous chemistry of monocarboxylic acids on α-Al2O3 at different relative humidities. Atmos. Chem. Phys..

[66] Wenzel R.N. (1936). Resistance of solid surfaces to wetting by water. Ind. Eng. Chem..

[67] Cassie A.B.D., Baxter S. (1944). Wettability of porous surfaces. Trans. Faraday Soc..

[68] Yu J., Zhao X., Yu J.C., Zhong G., Han J., Zhao Q. (2001). The grain size and surface hydroxyl content of super-hydrophilic TiO_2_/SiO_2_ composite nanometer thin films. J. Mater. Sci. Lett..

[69] Simonsen M.E., Li Z., Søgaard E.G. (2009). Influence of the OH groups on the photocatalytic activity and photoinduced hydrophilicity of microwave assisted sol–gel TiO_2_ film. Appl. Surf. Sci..

[70] Yu J., Zhao X., Zhao Q., Wang G. (2001). Preparation and characterization of super-hydrophilic porous TiO_2_ coating films. Mater. Chem. Phys..

[71] Balchev I., Minkovski N., Marinova T., Shipochka M., Sabotinov N. (2006). Composition and structure characterization of aluminum after laser ablation. Mater. Sci. Eng. B.

[72] Dabek-Zlotorzynska E., McGrath M. (2000). Determination of low-molecular-weight carboxylic acids in the ambient air and vehicle emissions: A review. Fresenius’ J. Anal. Chem..

[73] Grönberg L., Shen Y., Jönsson J.Å. (1993). Ion chromatographic determination of carboxylic in air with on-line liquid membrane pretreatment. J. Chromatogr. A.

